# Direct Asymmetric Reductive Amination for the Synthesis of (*S*)-Rivastigmine

**DOI:** 10.3390/molecules23092207

**Published:** 2018-08-31

**Authors:** Guorui Gao, Shaozhi Du, Yang Yang, Xue Lei, Haizhou Huang, Mingxin Chang

**Affiliations:** 1 College of Chemistry, Chemical Engineering and Materials Science, Collaborative Innovation Center of Functionalized Probes for Chemical Imaging, Shandong Normal University, 88 Wenhuadong Road, Jinan 250014, China; gaoguorui2001@163.com (G.G.); 17853135536@163.com (X.L.); 2 Shanxi Key Laboratory of Natural Products & Chemical Biology, College of Chemistry & Pharmacy, Northwest A&F University, 22 Xinong Road, Yangling, Shanxi 712100, China; m17792625262@163.com (S.D.); yy940331@outlook.com (Y.Y.)

**Keywords:** Alzheimer’s syndrome, rivastigmine, asymmetric reductive amination, asymmetric catalysis, phosphoramidite ligands

## Abstract

In this article we demonstrate how asymmetric total synthesis of (*S*)-rivastigmine has been achieved using direct asymmetric reductive amination as the key transformation in four steps. The route started with readily available and cheap *m*-hydroxyacetophenone, through esterification, asymmetric reductive amination, *N*-diphenylmethyl deprotection and reductive amination, to provide the final (*S*)-rivastigmine in 82% overall yield and 96% enantioselectivity. In the asymmetric reductive amination, catalysed by the iridium–phosphoramidite ligand complex and helped by some additives, the readily prepared 3-acetylphenyl ethyl(methyl)carbamate directly reductively coupled with diphenylmethanamine to yield the chiral amine product in 96% *ee* and 93% yield.

## 1. Introduction

**Figure 1:**
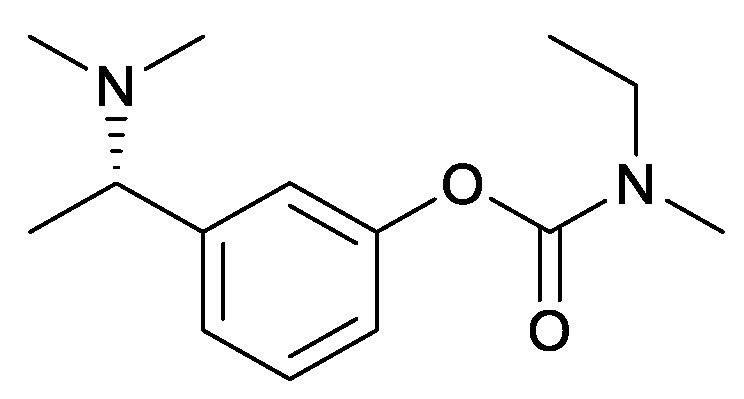
(*S*)-rivastigmine.

Alzheimer’s disease (AD) is the most common form of dementia. It is characterized by progressive loss of memory and other cognitive functions [1,2,3,4]. It is a severe human health threat with more than 40 million sufferers worldwide, and this number is expected to triple by 2050 [5,6]. Rivastigmine {3-[1-(dimethylamino)-ethyl]phenylethyl (methyl)carbamate)} (Figure 1) is the active pharmaceutical ingredient of Exelon that was developed for the treatment of patients with mild to moderate Alzheimer disease and treatment of dementia caused by Parkinson’s disease and Lewy bodies [7,8,9,10]. Only the (*S*)-enantiomer exhibits the desired biological activity.

To satisfy the demand of the enantiopure rivastigmine, several preparing routes have been developed. Among these, the method of racemate resolution using tartaric acid derivative is applied in the industrial production of (*S*)-rivastigmine [11]. Charette et al. reported a novel ligand, based on the bis(phosphine) monoxide framework, along with copper for the addition of Me_2_Zn to imine to build the key chiral motif [12]. A strategy of copper-catalysed stereoselective hydroamination reactions of alkynes was applied to prepare (*S*)-rivastigmine by S.L. Buchwald’s group [13]. This synthetic route was concise, but the starting material 3-ethynylphenol was expensive. List group utilized the asymmetric hydrogenation of the *N*-methyl imine as the core reaction to yield the drug molecule in four steps [14]. M.H. Xie’s group obtained the product in high enantiopurity via six steps and with expensive chiral amine as the starting material [15]. The method of chemoenzymatic asymmetric synthesis was also reported [16,17,18,19]. One scalable route is documented by Che et al. utilizing the asymmetric hydrogenation of corresponding ketone, in which toxic methanesulfonyl chloride was used [20]. Efficient and practical asymmetric synthesis of rivastigmine is still highly desired. Recently we have demonstrated that diphenylmethylamine is a universal amine source for the direct asymmetric reductive amination (DARA) of various ketones, catalyzed by iridium and readily available phosphoramidite ligand based on BINOL [21,22,23]. Here we report the convenient synthesis of (*S*)-rivastigmine applying the DARA strategy. In DARA, the polar carbamate group on the ketone substrate is well tolerated; the applied chiral ligands were easily prepared from inexpensive BINOL and displayed excellent reactivity and stereoselectivity.

## 2. Results and Discussion

**Scheme 1:**
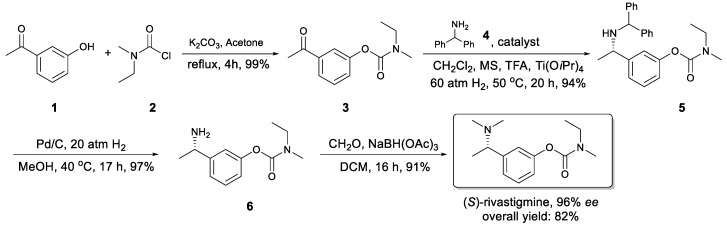
The synthetic route of (*S*)-rivastigmine.

Starting from readily available and cheap *m*-hydroxyacetophenone **1**, (*S*)-rivastigmine was synthesized in four steps in high yields and *ee* (Scheme 1).

In the first step, esterification between compound **1** and **2** was carried out under mild conditions to afford **3** in quantitative yield, which is the key intermediate and substrate for next step. On the basis of our group’s early work [21,22,23], the catalytic system of iridium–monodentate phosphoramidite and a few additives were adopted. The monodentate phosphoramidite chiral ligands are based on BINOL back-bone, which is a cheap and readily available bulk chemical. This kind of ligands is easily prepared, air-stable and well documented in a variety of catalytic reactions [24,25,26,27].

**Figure 2:**
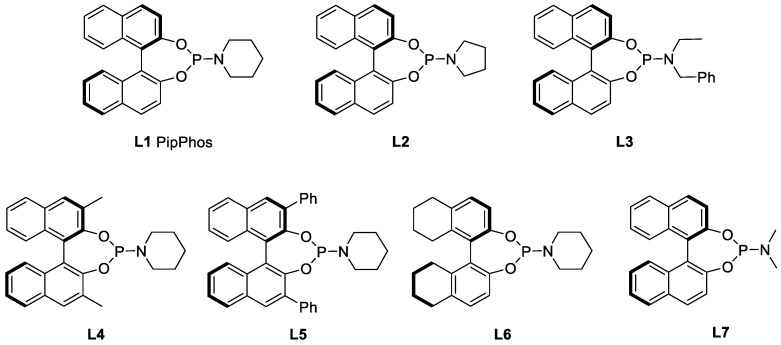
Structures of screened chiral ligands.

Using DCM as the solvent and PipPhos (Figure 2) as the chiral ligand (Table 1, entry 1), the reaction provided compound **5** in 96% *ee* and 84% yield. (^1^H & ^13^C NMR and HPLC spectra in Supplementary Materials). Common anion additives did not positively affect the reaction (Table 1, entries 2–3) [28,29,30]. With the addition of more TFA, both the stereoselectivity and the yield were improved (Table 1, entry 4). Several other Brønsted acids were tested and the results were not as satisfied as that from TFA (Table 1, entries 5–8). The above results indicated that TFA was crucial in this reaction. Next, a variety of phosphoramidite-type chiral ligands **L2**–**L6** (see Supplementary Materials for the general synthetic procedure) were explored (entries 9–13). Compared with **L1**, **L2** afforded similar *ee* but lower yield (entry 9). The non-cyclic amine moiety on **L3** did not function well (entry 10). More steric hindered ligands **L4** and **L5** led to poor reactivity (entries 11–12). These results indicate that both the amine moiety and the substituents on the back-bone of the ligands affected their reactivity dramatically. The H8-BINOL-based **L6** furnished slightly lower enantiomeric excess than **L1** (entry 13). The commercially available MonoPhos was also examined, and it provided moderate to good yield and enantiopurity (entry 14).

Using Ir–**L1** as the reaction catalyst, we further screened other reaction parameters. It is commonly believed that Ti(O*i*Pr)_4_ can promote the formation of the imine intermediate during the reductive amination process [31]. Our studies also indicated that the amount of tetraisopropoxytitanium displayed a great influence on the reaction. Especially, with the addition of 30%, compound **5** could be obtained in 96% *ee*. More Ti(O*i*Pr)_4_ had a negative effect on the enantioselectivity (Table 2, entry 3). As described above, TFA was important for this reaction. It could greatly improve the reactivity (entries 2–7, yield from 86% to 94%) as well as the enantioselectivity (*ee* from 96% to 97%). When the H_2_ pressure was decreased to 50 atm, the reaction *ee* remained the same but the yield dropped to 90% (entry 6). Further lowering the pressure to 30 atm, the yield for the reaction dropped to 75% but the enantioselectivity increased slightly to 98% (entry 7). When the catalyst loading was decreased to 0.5 mol%, the *ee* and yield of the reaction remained the same; further decreasing the catalyst loading to 0.2 mol% the reaction yield dropped slightly to 88% with *ee* at 96%; at 60 °C with the catalyst loading at 0.1 mol%, the reaction afforded 70% product. In comparison, **L6** outperformed **L1** under lower catalyst loading at 0.1 mol%, providing **5** in perfect yield and stereoselectivity (entry 11). Therefore, the optimal reaction conditions (entry 5) for the synthesis of compound **5** were determined based on enantioselectivity, yield and the cost of synthesis.

**Scheme 2:**
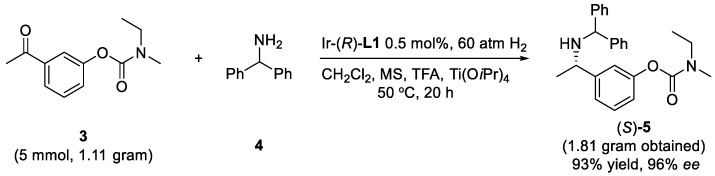
Gram-scale synthesis of (*S*)-**5**.

**Scheme 3:**
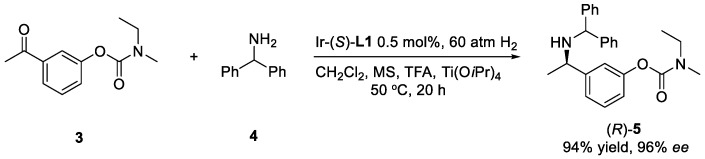
Application of (*S*)-**L1** for the synthesis of (*R*)-**5**.

To demonstrate the practical application of this protocol, the asymmetric reductive amination of **3** with **4** was carried out on large scale. The key intermediate **5** was obtained with 93% isolated yield and 96% *ee* (Scheme 2). Applying (*S*)-**L1** instead of (*R*)-**L1**, the corresponding (*R*)-5 was obtained in the same high yield and *ee* (Scheme 3), which demonstrates the versatility of asymmetric catalysis. The facile removal of the diphenylmethyl group was carried out with Pd/C as the catalyst and H_2_ as the reductant leading to primary amine product **6** in 97% yield, without any erosion of the enantioselectivity (Scheme 1).

Finally, (*S*)-rivastigmine was obtained through reductive amination of compound **6** with formaldehyde in the presence of sodium triacetoxyborohydride in CH_2_Cl_2_ at room temperature. It was purified via column chromatograph to render the pure product in 96% *ee* and 91% yield. Again, in this step the enantiopurity of the final product was not affected. Through this 4-step procedure, the final product (*S*)-rivastigmine was synthesized in 82% overall yield and 96% *ee*. Compared with common (*S*)-rivastigmine synthetic methods, this procedure is very efficient in terms of operational simplicity and scalability.

**Scheme 4:**
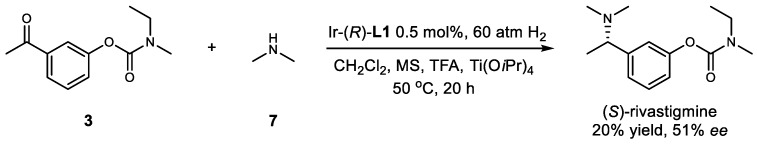
Application of dimethylamine for the synthesis of (*S*)-rivastigmine.

The direct asymmetric reductive amination of **3** with dimethylamine **7** was also investigated. Unfortunately, poor yield and moderate *ee* were achieved (Scheme 4).

## 3. Materials and Methods

### 3.1. Materials

Ethyl acetate (ACS grade), hexanes (ACS grade), methanol (ACS grade) and anhydrous dichloromethane for DARA (ACS grade) were obtained commercially and used without further purification. Toluene and tetrahydrofuran were purified according to standard methods unless otherwise noted. Commercially available reagents were used without further purification. Reactions were monitored by thin layer chromatography (TLC) using silicycle pre-coated silica gel plates (Qingdao Haiyang Chemical Co., Qingdao, China). Flash column chromatography was performed over silica gel (300–400 mesh).

### 3.2. Characterization

Mass spectra were recorded with Micromass QTOF2Quadrupole/Time-of-Flight Tandem mass spectrometer (Milford, MA, USA) using electron spray ionization. ^1^H-NMR spectra were recorded on a Bruker AV-400 spectrometer (Bruker, Fällanden, Switzerland) and a Bruker AV-500 (Bruker, Fällanden, Switzerland) spectrometer in chloroform-d. Chemical shifts are reported in ppm with the internal TMS signal at 0.0 ppm as a standard. The data is being reported as (s = singlet, d = doublet, dd = doublet of doublets, t = triplet, dt = doublet of triplets, m = multiplet or unresolved, q = quartet, dq = doublet of quartets, brs = broad singlet, coupling constant(s) in Hz, integration). ^13^C-NMR spectra were recorded on a Bruker AV-400 spectrometer and a Bruker AV-500 spectrometer in chloroform-d. Chemical shifts are reported in ppm with the internal chloroform signal at 77.0 ppm as a standard.

### 3.3. Preparation of Compound ***3***

To a solution of compound **1** (5.11 g, 38 mmol) in acetone (100 mL) was added K_2_CO_3_ (10.44 g, 76 mmol) and compound **2** (7.20 g, 58 mmol) subsequently. Then the solution was heated to reflux for 4h. After the reaction solution was cooled to r.t., it was filtered and washed with acetone (30 mL × 3). Then the filtrate was concentrated under reduced pressure and purified via column chromatography (CH_2_Cl_2_/MeOH = 40:1 to 20:1 *v*/*v*) to afford light yellow oil (Yield: 100%). ^1^H-NMR (500 MHz, Chloroform-d): δ 7.79 (d, *J* = 8.0 Hz, 1H, Ar-H), 7.69 (s, 1H, Ar-H), 7.47 (t, *J* = 7.5 Hz, 1H, Ar–H), 7.35 (d, *J* = 2.5 Hz, 1H, Ar–H), 3.50 (dq, *J* = 7.5, 14.5 Hz, 2H, –CH_2_CH_3_), 3.08 (d, *J* = 42.5 Hz, 3H, –NCH_3_), 2.59 (s, 3H, –COCH_3_), 1.27 (dt, *J* = 7.0 Hz, *J* = 7.0 Hz, 3H, –CH_2_CH_3_).

### 3.4. Preparation of Compound ***5***

Compound **3** (0.2 mmol), **4** (0.26 mmol, 1.3 equiv.) and TFA (0.8 equiv.) in CH_2_Cl_2_ were added to a small vial, followed by Ti(O*i*Pr)_4_ (0.06 mmol, 0.3 equiv.) and the Ir–**L1** (1 mol%) solution in CH_2_Cl_2_, which was in situ generated from stirring the solution of [Ir(cod)Cl]_2_ and **L1** in CH_2_Cl_2_ for 20 min. The resulting vial was transferred to an autoclave, which was charged with 60 atm of H_2_, and stirred at 50 °C for 20 h. The reaction was quenched with aqueous sodium bicarbonate solution and extracted with CH_2_Cl_2_ (2 mL × 3). The organic phase was dried over anhydrous Na_2_SO_4_, concentrated and purified by column chromatography (EtOAc/Hex) to give the chiral amine product **5**, which was analyzed by chiral HPLC determine the enantiomeric excess (Yield: 93%, *ee*: 96%). ^1^H-NMR (500 MHz, Chloroform-d): δ 7.41–7.36 (m, 7H, ArH), 7.33–7.30 (m, 3H, ArH), 7.25 (t, *J* = 6.5 Hz, 1H, ArH), 7.14 (d, *J* = 6.5 Hz, 1H, ArH), 7.09 (s, 2H, ArH), 4.75 (s, 1H, –CH(Ph)_2_), 3.76 (d, *J* = 6.0 Hz, 1H, –CHCH_3_C_6_H_4_–), 3.54 (dd, *J* = 5.5, 2H, –CH_2_CH_3_), 3.13 (d, *J* = 35 Hz, 3H, –NCH_3_), 1.44 (d, *J* = 6.5 Hz, 3H, –CHCH_3_), 1.31–1.27 (m, 3H, –CH_2_CH_3_).

### 3.5. Preparation of Compound ***6***

Compound **5** (0.2 mmol, 78 mg), Pd/C (8 mg, 10%, 50% wetted with water) and MeOH (2 mL) were added to a vial. The resulting vial was transferred to an autoclave, which was charged with 20 atm of H_2_, and stirred at 40 °C for 17 h. The hydrogen gas was released slowly and the solution was filtered to removed Pd/C. The filtrate was concentrated and purified by flash column chromatography (EtOAc/Hex) to yield the desired product **6** (43 mg, yield: 97%). ^1^H-NMR (500 MHz, Chloroform-d): δ 7.34–7.31 (m, 1H, ArH), 7.20–7.14 (m, 2H, ArH), 7.01 (s, 1H, ArH), 4.14 (d, *J* = 6.0 Hz, 1H, –CHCH_3_), 3.50 (dd, *J* = 6.0 Hz, *J* = 6.0 Hz, 2H, –CH_2_CH_3_), 3.09 (d, *J* = 38 Hz, 3H, –NCH_3_), 1.64 (s, 2H, –NH_2_), 1.41 (d, *J* = 7.0 Hz, 3H, –CHCH_3_), 1.27 (d, *J* = 26 Hz, 3H, –CH_2_CH_3_).

### 3.6. Preparation of (S)-Rivastigmine

To a solution of compound **6** (64 mg, 0.29 mmol) in CH_2_Cl_2_ (6 mL), Na_2_SO_4_ (42 mg, 0.30 mmol), NaBH(OAc)_3_ (504 mg, 2.4 mmol) and formaldehyde (37% in water, 88 μL, 1.0 mmol) were added subsequently. Then the reaction mixture was stirred at −10 °C for 8 h. The reaction was quenched with aqueous K_2_CO_3_ solution. The phases were separated, the aqueous phase was extracted with EtOAc (20 mL × 2) and the combined organic phase was dried over Na_2_SO_4_, filtered and concentrated under reduced pressure to give crude product, which was purified by flash column chromatography (DCM/MeOH = 20:1) to yield the desired (*S*)-Rivastigmine as oil (Yield: 91%, *ee*: 96%). [α]${}_{D}^{25}$ = −29.8 (c = 1.0, EtOH) ([α]${}_{D}^{29}$ = −32.8 (c = 1.3, EtOH))[16]. ^1^H-NMR (400 MHz, Chloroform-d): δ 7.31–7.27 (m, 1H, ArH), 7.12 (d, *J* = 9.5 Hz, 1H, ArH), 7.06 (s, 1H, ArH), 7.02 (d, *J* = 10.0 Hz, 1H, ArH), 3.49–3.38 (m, 2H, –CH_2_CH_3_), 3.27 (q, *J* = 8.5 Hz, *J* = 16.5 Hz, 1H, –CHCH_3_), 3.06 (d, *J* = 37 Hz, 3H, –NCH_3_), 2.22 (s, 6H, –N(CH_3_)_2_), 1.37 (d, *J* = 8.5 Hz, 3H, –CHCH_3_), 1.25–1.17 (m, 3H, –CH_2_CH_3_); ^13^C-NMR (100 MHz, Chloroform-d): 153.59, 150.54, 144.72, 127.85, 123.20, 119.74, 64.62, 43.01, 42.18, 33.18, 19.04, 12.21. HRMS for C_14_H_23_N_2_O_2_: [M + H]^+^ *m*/*z* 251.17540, found *m*/*z* 251.17538.

## 4. Conclusions

In conclusion, (*S*)-rivastigmine (96% *ee*) was synthesized *via* a 4-step route in 82% overall yield, which (to the best of our knowledge) depicts the highest yield route to enantiopure rivastigmine reported to date. In this route, we utilized the highly efficient direct asymmetric reductive amination (DARA) to provide the key intermediate compound **5** in excellent enantioselectivity and yield from the corresponding ketone. DARA is featured with high atom-efficiency and operational simplicity. The chiral ligands we used in the DARA step are easily modulated and readily prepared from inexpensive starting materials. Our strategy opens the door for the practical catalytic asymmetric production of (*S*)-rivastigmine.

## Figures and Tables

**Table 1:**
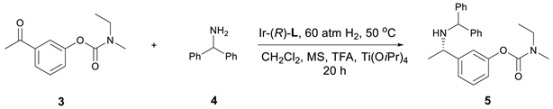
The screening of reaction conditions ^a^.

Entry	Ligand	Acid (Equiv.)	Yield (%)	*ee* (%)
1	**L1**	TFA (0.5)	84	96
2 ^b^	**L1**	TFA (0.5)	76	96
3 ^c^	**L1**	TFA (0.5)	45	77
4	**L1**	TFA (1.0)	93	96
5	**L1**	4-Cl-C_6_H_4_SO_3_H (1.0)	<15	-
6	**L1**	TsOH (1.0)	<15	-
7	**L1**	4-NO_2_-C_6_H_4_CO_2_H (1.0)	65	5
8	**L1**	CCl_3_CO_2_H (1.0)	77	92
9	**L2**	TFA (1.0)	60	90
10	**L3**	TFA (1.0)	16	58
11	**L4**	TFA (1.0)	<10	-
12	**L5**	TFA (1.0)	<10	-
13	**L6**	TFA (1.0)	94	95
14	**L7**	TFA (1.0)	78	82

^a^ Reaction conditions: [Ir]/L/**3**/**4** = 1:2.2:100:130, **3** 0.1 mmol, CH_2_Cl_2_ 2 mL, 60 atm of H_2_, 50 °C, 20 h; MS = molecular sieves, 0.1 gram; TFA = trifluoroacetic acid; Ti(O*i*Pr)_4_ 0.2 equiv.; Yields were isolated yields; Enantiomeric excesses were determined by chiral HPLC (see Supplementary Materials for details). ^b^ 10 mol% NaBF_4_ was added. ^c^ 10 mol% NH_4_I was added.

**Table 2:**
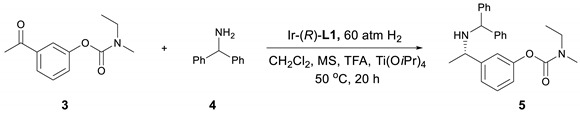
The screening of the amount of the additives ^a^.

Entry	Catalyst Loading (mol%)	Ti(O*i*Pr)_4_ (mol%)	Acid (mol%)	Yield (%)	*ee* (%)
1	1	20	50	79	93
2	1	30	50	85	96
3	1	40	50	87	94
4	1	30	70	91	96
5	1	30	80	94	97
6 ^b^	1	30	80	90	97
7 ^c^	1	30	80	75	98
8	0.5	30	80	94	96
9	0.2	30	80	88	96
10 ^d^	0.1	30	80	70	94
11 ^e^	0.1	30	80	94	95

^a^ Reaction conditions: Catalyst loading is based on the [Ir] monomer, 1 mol%; **3** 0.2 mmol, CH_2_Cl_2_ 2 mL, 50 °C, 20 h; MS = molecular sieves, 0.2 gram; TFA = trifluoroacetic acid; Yields were isolated yields; Enantiomeric excesses were determined by chiral HPLC. ^b^ The H_2_ pressure was 50 atm. ^c^ The H_2_ pressure was 30 atm. ^d^ The reaction temperature was 60 °C. ^e^ The applied chiral ligand was **L6**.

## Supplementary Materials

Supplementary Materials are available online, spectra for all products and HPLC for intermediate 5 and (*S*)-rivastigmine.
