# Supplementary material for: Direct Asymmetric Reductive Amination for the Synthesis of (S)-Rivastigmine
Source: Molecules. 2018 Aug 31;23(9):2207. doi: 10.3390/molecules23092207 (PMC6225309; doi:10.3390/molecules23092207)
Supplement: Supplementary file 1 [file molecules-23-02207-s001.pdf]

Supporting information

# Direct Asymmetric Reductive Amination for the Synthesis of (*S*)-Rivastigmine

Guorui Gao [1,#], Shaozhi Du [2,#], Yang Yang [2], Xue Lei [1], Haizhou Huang [2,*], and Mingxin Chang[2,*]

1 College of Chemistry, Chemical Engineering and Materials Science, Collaborative Innovation Center of Functionalized Probes for Chemical Imaging in Universities of Shandong, Shandong Normal University, 88 Wenhuadong Road, Jinan 250014, PR China; gaoguorui2001@163.com

2 Shanxi Key Laboratory of Natural Products & Chemical Biology, College of Chemistry & Pharmacy, Northwest A&F University, 22 Xinong Road, Yangling, Shanxi 712100, PR China Phone/fax: (+86)-29-8709-2662; mxchang@nwsuaf.edu.cn

* Correspondence: huanghai30@163.com; mxchang@nwsuaf.edu.cn; Tel.: +86-29-8709-2662

**CONTENTS**

I The synthetic route of (S)-rivastigmine S2
II General Procedure for Preparation of Monophos-type Ligands S2
III References S3
IV NMR & Chiaral HPLC & HRMS Spectra S4

*Molecules* **2018**, *23*, 2207; doi:10.3390/molecules23092207

## I. The synthetic route of (S)-rivastigmine

(S)-Rivastigmine was prepared according the scheme 1 below

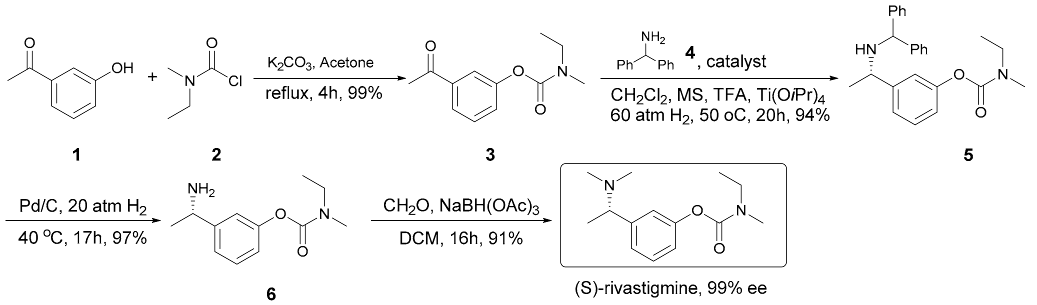


Scheme 1. the synthetic route of (S)-rivastigmine

## II. General Procedure for Preparation of Monophos-type ligands

Ligands **L1, L2, L3** were synthesized according to the reported procedures[1-7].

1)

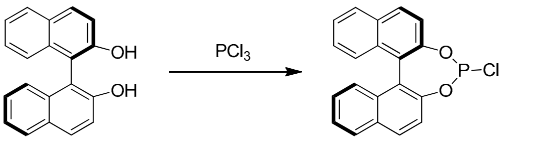


**L1-1** **L1-2**

A 25 mL Schlenk flask was charged with (*R*)-(+)-1,1-bi(2-naphthol) (0.57g, 2 mmol), phosphorus trichloride (2.74 g, 20 mmol, 10 equiv), 1-methyl-2-pyrrolidinone (1.6 μL, 0.02 mmol, 0.008 equiv) under nitrogen. The reaction mixture was heated to 90 oC for 15 min, and all volatiles were removed under reduced pressure. CH2Cl2 (2 mL×2) was used to remove the traces of phosphorus trichloride. The resulting oil was vacummed for 3 h to give the pale solid which was used directly in next step.

2)

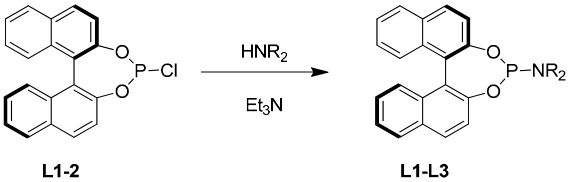


**L1-2** **L1-L3**

A 25 mL round-bottom flask was charged with 2 mmol of corresponding amine, 3 mmol of Et3N and 10 ml toluene. The above made chlorophosphite was dissolved in 5 mL toluene and was transfered to the reaction flask. The mixture was stirred for 3 h. The solid was removed by filtration. The filtrate was concentrated and purified by flash column chromatography (EtOAc /Hex) to yield desired ligand (yield: 75-95%).

Ligands **L4** were synthesized according to the reported procedures[5].

1)

*Molecules* **2018**, *23*, 2207; doi:10.3390/molecules23092207

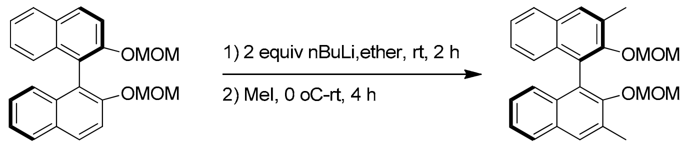


At room temperature, *n*-butyllithium (1.32 mL of a 2.5 M solution in hexanes, 3.3 mmol) was added to a a solution of (*S*)-2,2'-bis(methoxymethoxy)-1,1'-binaphthyl (0.62 g, 1.65 mmol) dissolved in anhydrous ether (25 mL). After 2 h, the resulting gray suspension was cooled to 0℃ and methyl iodide (0.47 g, 3.3 mmol) was added. The reaction mixture was warmed to room temperature and stirred for further 4 h, after which it was quenched with a saturated aqueous solution of $NH_4Cl$ (25 mL). The aqueous phase was extracted with ethyl acetate (3 × 25 mL) and the combined with the above organic phases which were washed with $H_2O$ (25 mL) and brine (25 mL). The solution was dried over $Na_2SO_4$, filtered, and concentrated *in vacuo*. The resulting crude reaction product was purified by chromatography (PE/EA=5:1) to afford **L4-2** as a white powder (72% yield).

2)

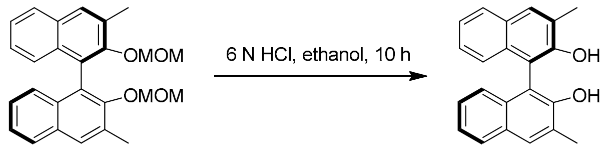


a 50-mL round bottom was charged with **L4-2** (0.35 g, 1.25 mmol). The reagent was dissolved in a minimum amount of $CH_2Cl_2$ (2 mL) and ethanol (7.0 mL) and 6 N HCl (7.0 mL) were added successively. The mixture was then heated at reflux for 10 h and the resulting yellow solution was concentrated *in vacuo* after cooling to room temperature. Water (10 mL) was added and the solution was extracted with $CH_2Cl_2$ (5 × 12 mL), after which the organic layers were combined and dried over $Na_2SO_4$. The drying agent was removed via filtration and the filtrate was concentrated *in vacuo*. The remaining crude product was purified by column chromatography (PE/EA= 5:1) to give **L4-3** as a white powder (80% yield).

3) The following procedure is the same as for the synthesis of **L1-3**.

Ligands **L6** were synthesized according to the reported procedures [1-7].

1) (R)-(+)-1,1-bi(2-naphthol) (0.57g, 2 mmol) 10 % Pd/C (0.12 g, 50 % wet) and 10 mL of ethanol were placed into a 50 mL autoclave and stirred under 50 bar $H_2$ at 70℃ for 16 h. The reaction mixture was cooled to rt, Pd/C was filtered off and washed with ethanol (3x5 mL). The combined filtrates were concentrated in vacuum to give 0.588 g of H8-BINOL (yield: 100%).

2) The following procedure is the same as for the synthesis of **L1-L3**.

## III References

1. Huang, H.; Liu, X.; Zhou, L.; Chang, M.; Zhang, X. Direct asymmetric reductive amination for the synthesis of chiral β-arylamines. *Angew. Chem. Int. Ed.* **2016**, 55, 5309-5312, 10.1002/anie.201601025.

2. Lefort, L.; Boogers, J.A.F.; de Vries, A.H.M.; de Vries, J.G. Instant Ligand Libraries. Parallel Synthesis of Monodentate Phosphoramidites and in Situ Screening in Asymmetric Hydrogenation.

*Molecules* **2018**, *23*, 2207; doi:10.3390/molecules23092207

*Org. Lett.* **2004,** 6, 1733-1735, 10.1021/ol049510e.

3. Smith, C.R.; Mans, D.J.; RajanBabu, T.V., (R)-2,2'-binaphthoyl- (S,S)-di(1-phenylethyl) aminophosphine. Scalable protocols for the syntheses of phosphoramidite (feringa) ligands. *Org. Synth.* **2008**, 85, 238-247, 10.15227/orgsyn.085.0238.

4. Wang, B.; Feng, X.; Huang, Y.; Liu, H.; Cui, X.; Jiang, Y. A highly enantioselective hetero-Diels-Alder reaction of aldehydes with Danishefsky's diene catalyzed by chiral titanium(IV) 5,5,6,6,7,7,8,8-octahydro-1,1-bi-2-naphthol complexes. *J. Org. Chem*. **2002**, 67, 2175-2182, . 10.1021/jo016240u.

5. Graves, C.R.; Zhou, H.; Stern, C.L.; Nguyen, S.T. A mechanistic investigation of the asymmetric Meerwein-Schmidt-Ponndorf-Verley reduction catalyzed by BINOL/$AlMe_3$ - structure, kinetics, and enantioselectivity. *J. Org. Chem*. **2007**, 72, 9121-9133, 10.1021/jo070563u.

6. Zhao, W.; Wang, T.; Zhao, R.; Xie, H.; Liu, L. Enantioselective copper(II)-catalyzed conjugate addition of diethylzinc to β-substituted enones utilizing BINOL-based phosphoramidite ligands *Tetrahedron: Asymmetry*, **2016,** 27, 157-162, org/10.1016/j.tetasy.2016.01.016.

7. Cram, D.J., Helgeson, R.C., Peacock, S.C., Kaplan, L.J., Domeier, L.A., Moreau, P., Koga, K., Mayer, J.M.; Chao, Y.; Siegel, M.G.; Hoffman, D.H.; Sogah, G.D.Y. Host-Guest complexation. 8. macrocyclic polyethers shaped by two rigid substituted dinaphthyl or ditetralyl units. *J. Org. Chem*. **1978,** 43, 1930-1946, 10.1021/jo00404a019.

## IV NMR & Chiral HPLC & HRMS Spectra

$^{1}$H-NMR of **L4 (**500 MHz, Chloroform-d**):**

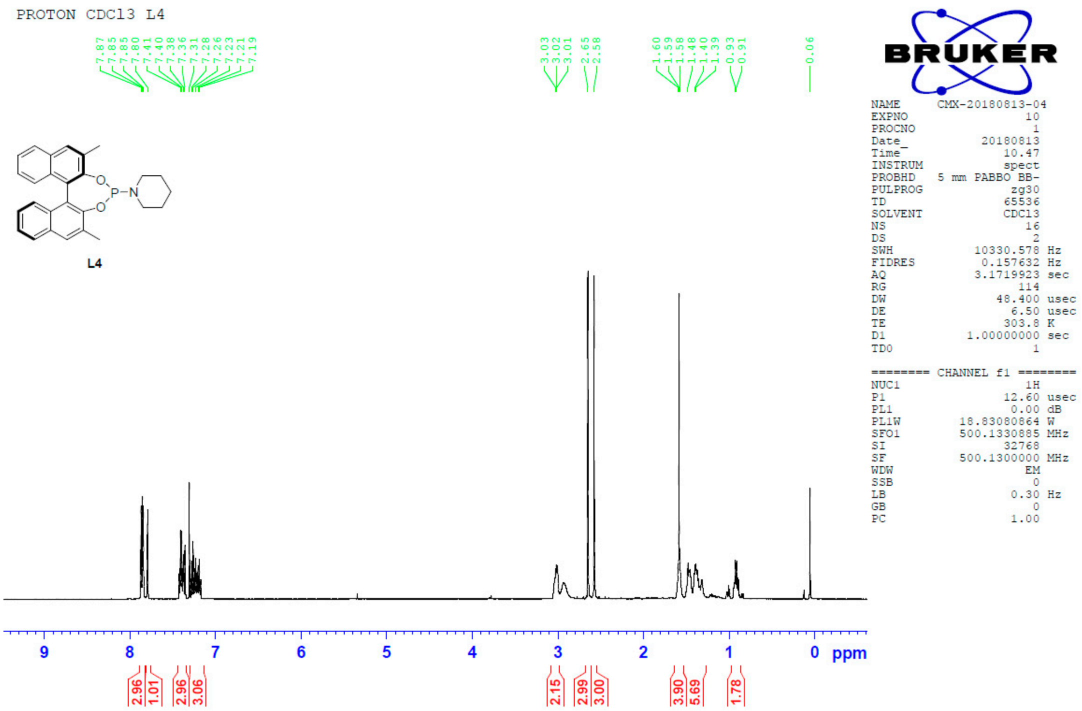


$^{31}$P-NMR of **L4 (**500 MHz, Chloroform-d**):**

*Molecules* **2018**, *23*, 2207; doi:10.3390/molecules23092207

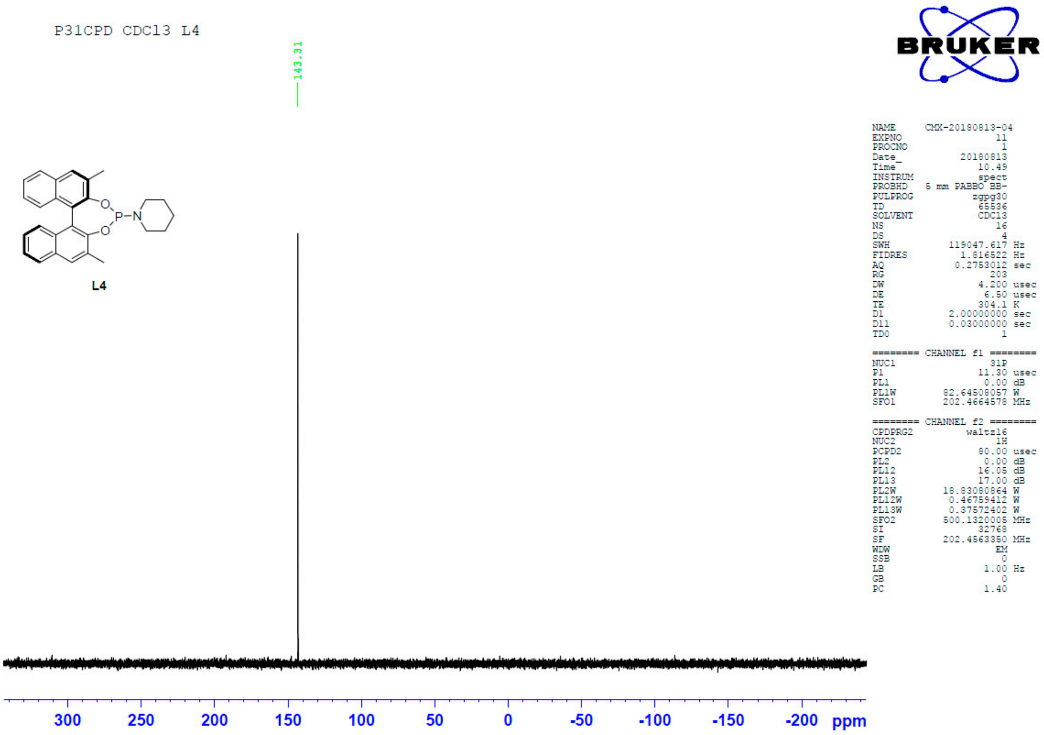


$^1$H-NMR of Compound **3(**500 MHz, Chloroform-d**):**

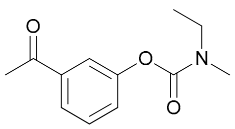

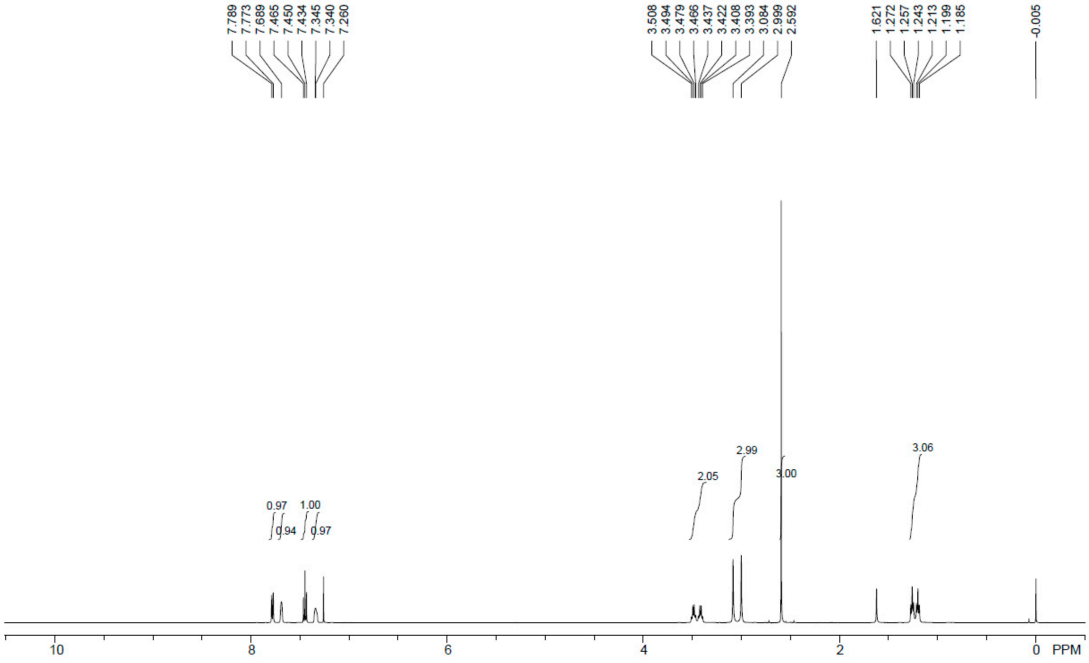

*Molecules* **2018**, *23*, 2207; doi:10.3390/molecules23092207

$^{1}$H-NMR of Compound **5(**500 MHz, Chloroform-d**):**

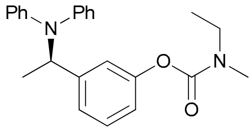


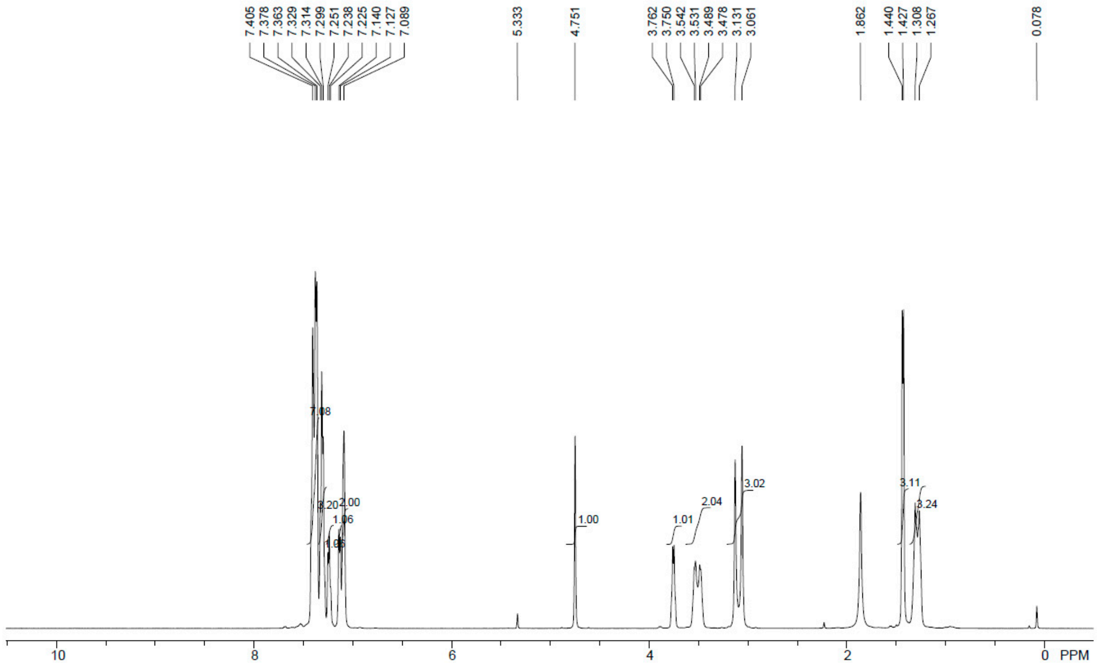

*Molecules* **2018**, *23*, 2207; doi:10.3390/molecules23092207

$^{13}$C-NMR of Compound **5** (125 MHz, Chloroform-d):

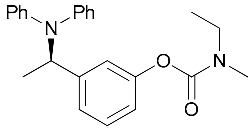


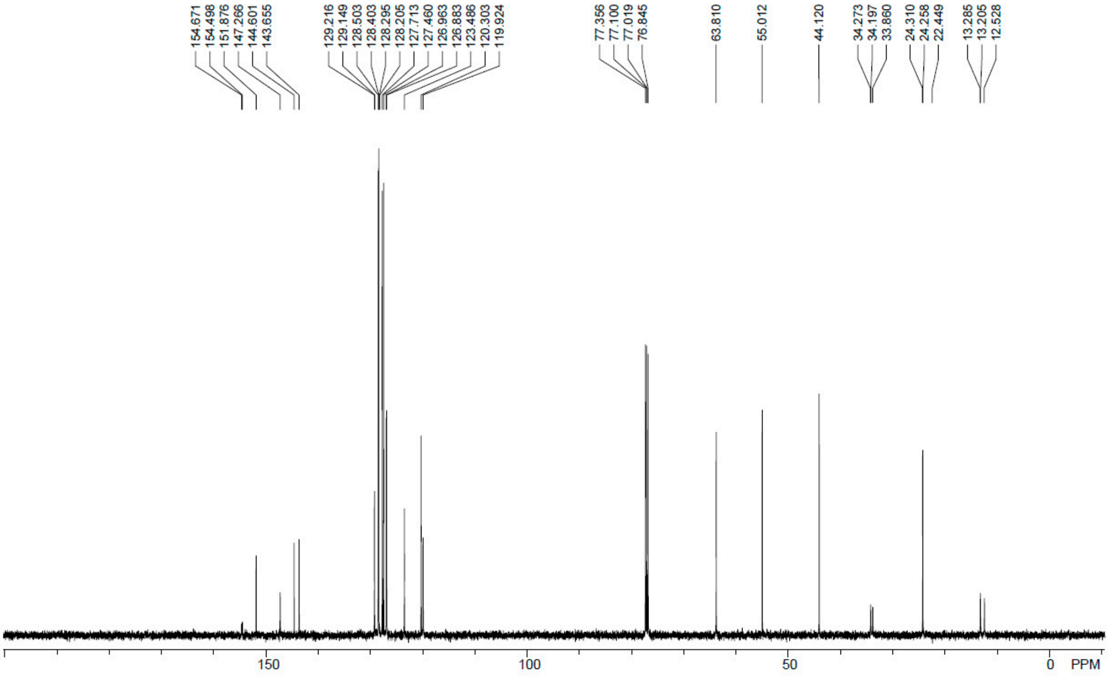


Chiral HPLC of compound rac **5** (Chiracel-OD, n-hexane/2-propanol=99.4/0.6, flow rate =0.9 mL/min,UV220 nm):

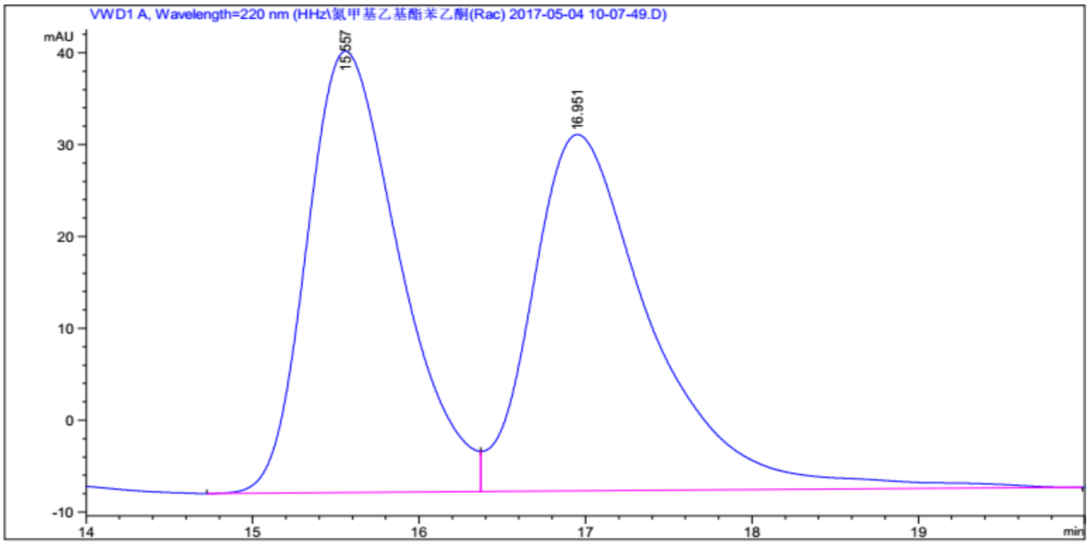

*Molecules* **2018**, *23*, 2207; doi:10.3390/molecules23092207

Chiral HPLC of compound **5** (Chiracel-OD, n-hexane/2-propanol=99.4/0.6, flow rate =0.9 mL/min,UV220 nm):

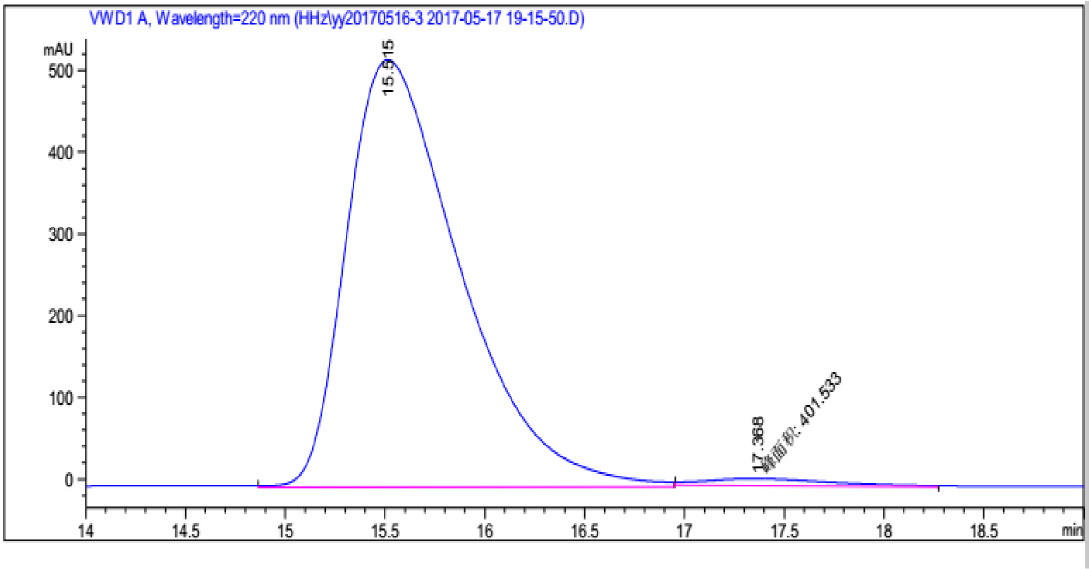

*Molecules* **2018**, *23*, 2207; doi:10.3390/molecules23092207

$^{1}$H-NMR of Compound **6** (500 MHz, Chloroform-d**):**

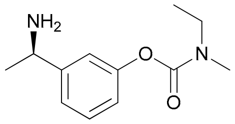


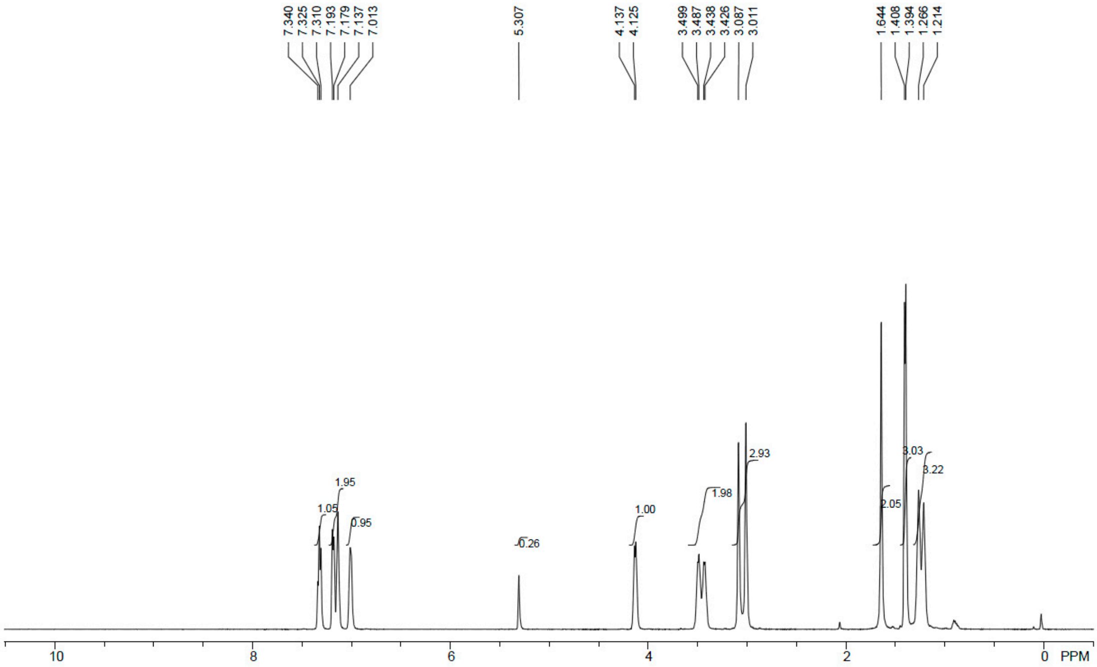


$^{1}$H-NMR of (S)-Rivastigmine**(**400 MHz, Chloroform-d**):**

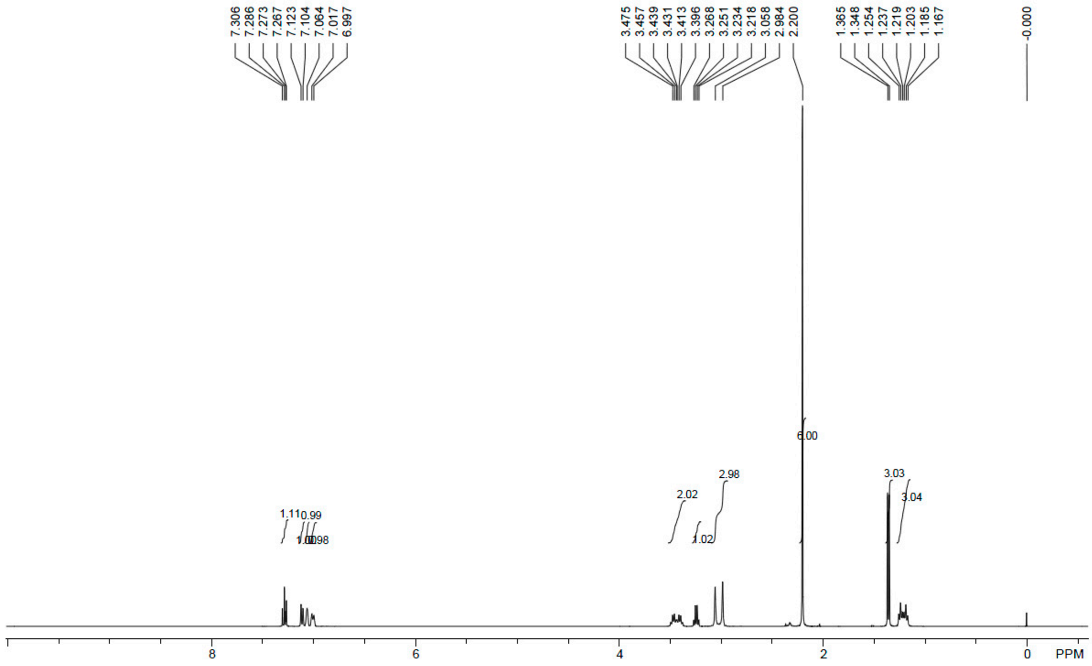

*Molecules* **2018**, *23*, 2207; doi:10.3390/molecules23092207

$^{13}$C-NMR of (S)-Rivastigmine **(**100 MHz, Chloroform-d**):**

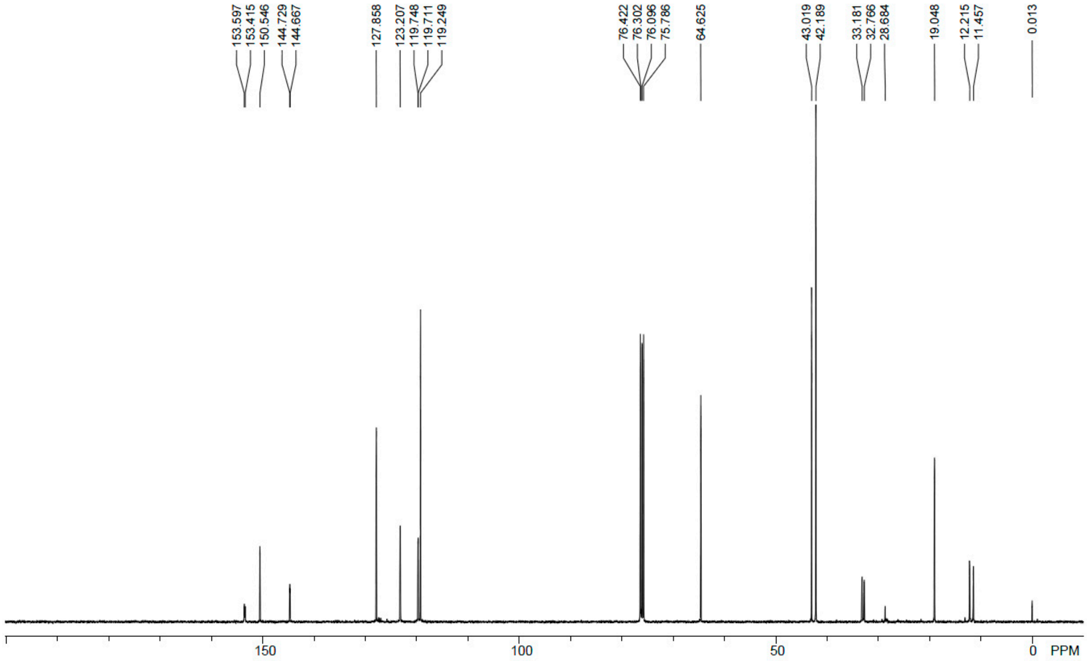

*Molecules* **2018**, *23*, 2207; doi:10.3390/molecules23092207

HRMS of (S)-Rivastigmine:

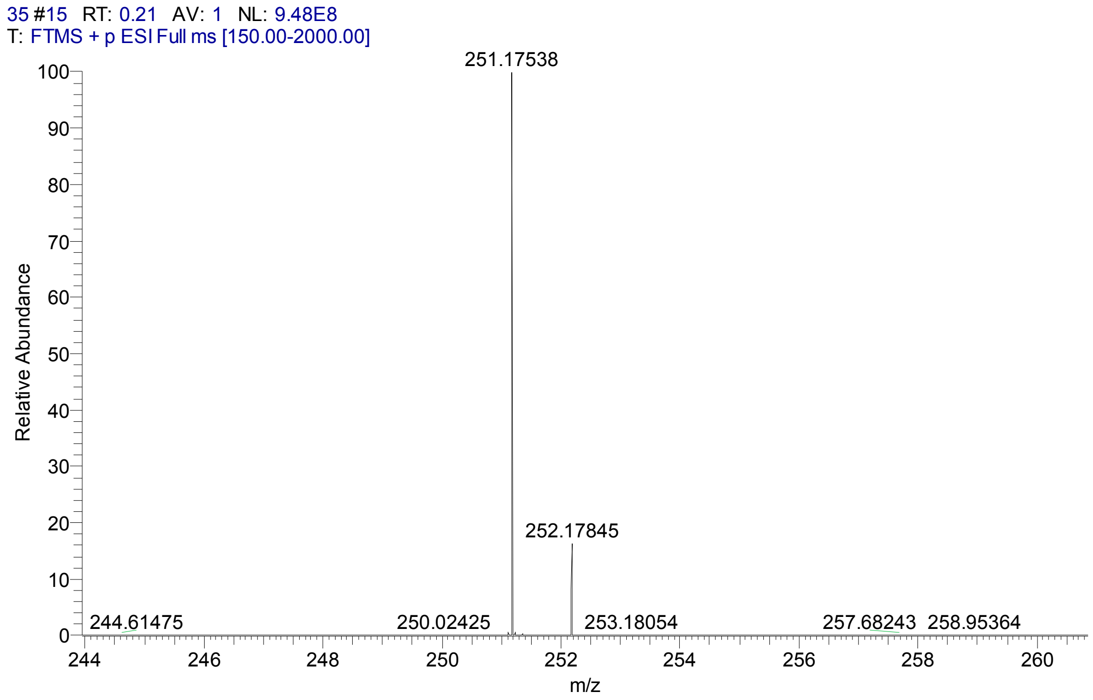

*Molecules* **2018**, *23*, 2207; doi:10.3390/molecules23092207

Chiral HPLC of Rivastigmine (Chiracel-OD, n-hexane/2-propanol/Methanol/Diethylamine =80/15/5/0.1, flow rate =1.0 mL/min,UV220 nm):

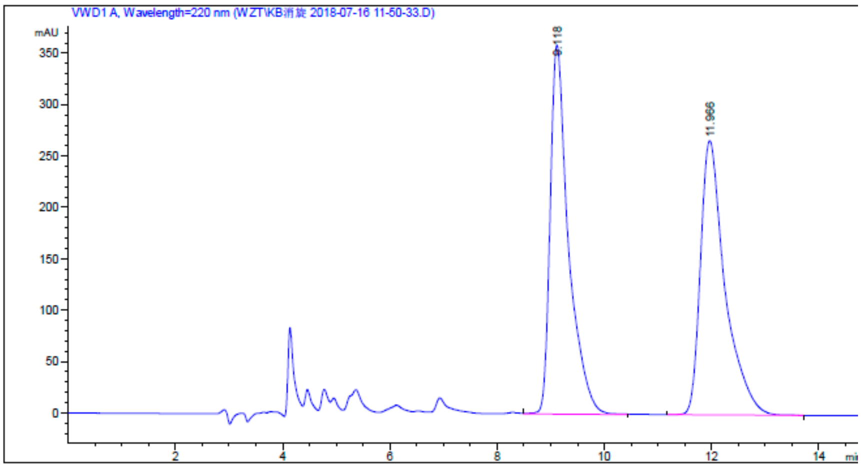


======================================================================

面积百分比报告

======================================================================

排序 : 信号
乘积因子 : 1.0000
稀释因子 : 1.0000
内标使用乘积因子和稀释因子

信号 1: VWD1 A, Wavelength=220 nm

| 峰 # | 保留时间 [min] | 类型 | 峰宽 [min] | 峰面积 [mAU*s] | 峰高 [mAU] | 峰面积 % |
|---|---|---|---|---|---|---|
| 1 | 9.118 | VV | 0.3466 | 8510.06250 | 358.91840 | 50.0618 |
| 2 | 11.966 | BB | 0.4690 | 8489.04004 | 266.96777 | 49.9382 |

*Molecules* **2018**, *23*, 2207; doi:10.3390/molecules23092207

Chiral HPLC of (S)-Rivastigmine (Chiracel-OD, n-hexane/2-propanol/Methanol/Diethylamine =80/15/5/0.1, flow rate =1.0 mL/min,UV220 nm):

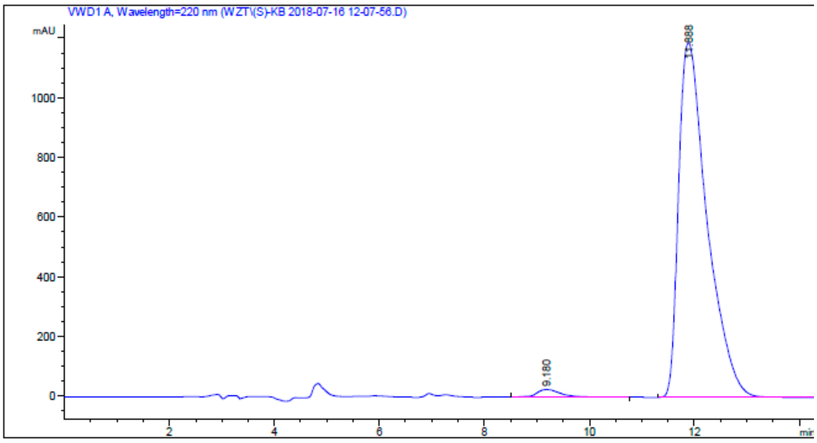


==================================================================

面积百分比报告

==================================================================

排序 : 信号
乘积因子 : 1.0000
稀释因子 : 1.0000
内标使用乘积因子和稀释因子

信号 1: VWD1 A, Wavelength=220 nm

| 峰 # | 保留时间 [min] | 类型 | 峰宽 [min] | 峰面积 [mAU*s] | 峰高 [mAU] | 峰面积 % |
|---|---|---|---|---|---|---|
| 1 | 9.180 | VV | 0.5142 | 869.88580 | 25.42498 | 1.9072 |
| 2 | 11.888 | BB | 0.5625 | 4.47403e4 | 1187.63635 | 98.0928 |
